# A novel polymer-based nitrocellulose platform for implementing a multiplexed microfluidic paper-based enzyme-linked immunosorbent assay

**DOI:** 10.1038/s41378-022-00385-z

**Published:** 2022-05-19

**Authors:** Dong Lin, Bowei Li, Longwen Fu, Ji Qi, Chunlei Xia, Yi Zhang, Jiadong Chen, Jaebum Choo, Lingxin Chen

**Affiliations:** 1 CAS Key Laboratory of Coastal Environmental Processes and Ecological Remediation; Shandong Key Laboratory of Coastal Environmental Processes, Yantai Institute of Coastal Zone Research, Chinese Academy of Sciences, 264003 Yantai, China; 2 School of Pharmacy, Binzhou Medical University, 264003 Yantai, China; 3 University of Chinese Academy of Sciences, 100049 Beijing, China; 4 Center for Ocean Mega-Science, Chinese Academy of Sciences, 266071 Qingdao, China; 5 Institute of Analytical Food Safety, School of Food Science and Technology, Jiangnan University, 214122 Wuxi, China; 6 Department of Chemistry, Chung-Ang University, Seoul, 06974 South Korea

**Keywords:** Chemistry, Materials science

## Abstract

Nitrocellulose (NC) membranes, as porous paper-like substrates with high protein-binding capabilities, are very popular in the field of point-of-care immunoassays. However, generating robust hydrophobic structures in NC membranes to fabricate microfluidic paper-based analytical devices (μPADs) remains a great challenge. At present, the main method relies on an expensive wax printer. In addition, NC membranes very easy to adhere during the printing process due to electrostatic adsorption. Herein, we developed a facile, fast and low-cost strategy to fabricate μPADs in NC membranes by screen-printing polyurethane acrylate (PUA) as a barrier material for defining flow channels and reaction zones. Moreover, hydrophobic barriers based on UV-curable PUA can resist various surfactant solutions and organic solvents that are generally used in immunoassays and biochemical reactions. To validate the feasibility of this PUA-based NC membrane for immunoassays in point-of-care testing (POCT), we further designed and assembled a rotational paper-based analytical device for implementing a multiplexed enzyme-linked immunosorbent assay (ELISA) in a simple manner. Using the proposed device under the optimal conditions, alpha fetoprotein (AFP) and carcinoembryonic antigen (CEA) could be identified, with limits of detection of 136 pg/mL and 174 pg/mL, respectively, which are below the threshold values of these two cancer biomarkers for clinical diagnosis. We believe that this reliable device provides a promising platform for the diagnosis of disease based on ELISA or other related bioassays in limited settings or remote regions.

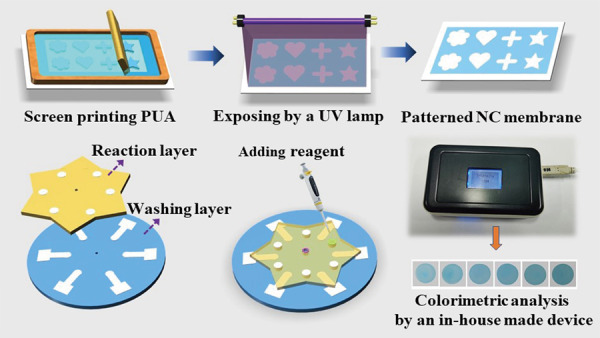

## Introduction

The enzyme-linked immunosorbent assay (ELISA) is a powerful tool for clinical diagnosis and healthcare monitoring due to its high specificity and sensitivity^1,2^. However, conventional ELISA is difficult to use in home medical care or remote regions due to the lack of trained professionals or appropriate laboratory conditions^3,4^. Thus, it is urgently necessary to develop an easy-to-operate technique to perform complex ELISAs. The lateral flow assay (LFA) has provided an inexpensive and rapid immunoassay strategy for the diagnosis of diseases or physiological conditions since it was initially commercially employed for female pregnancy assessment in the 1980s^5^. Despite their merit and multipurpose nature, LFAs are still considered to be able to provide only a qualitative analysis result with a high limit of detection (LOD).

Microfluidic paper-based analytical devices (μPADs) were first reported by the Whitesides group in 2007^6^. Since then, μPADs have demonstrated great potential as analysis platforms in point-of-care testing (POCT)^7,8^ and field testing^9,10^ due to their attractive features, such as affordable cost, low reagent consumption, portability and being driven without external force^11–13^. Combined with efficient sensing techniques, including colorimetric^14–16^, electrochemical^17,18^, fluorescence^19,20^ and chemiluminescent methods^21^, μPADs provide miniaturized integrated platforms for the development of immunoassays. Currently, microfluidic paper-based immunoassays have been applied to detect tumor markers, the influenza virus, malaria, and pathogenic bacteria^22–27^. However, most microfluidic paper-based immunoassay devices are made from pure cellulose paper, which is usually not considered an ideal substrate for the immobilization of biomolecules (e.g., proteins, DNA and RNA) because of the influence of the surface chemistry properties of the paper and its uneven porosity^28^.

Nitrocellulose (NC) membranes, which have high protein-binding capability, are well suited for protein immobilization-related applications, including LFAs, western blotting, dot ELISAs and nucleic acid testing^29–33^. In addition, compared with pure cellulose paper, this material possesses a more uniform pore size and smoother surface, which is critical to obtain stable and reproducible testing results. Lin et al. first reported the fabrication of μPADs in a NC membrane by the wax printing method, and the developed device was successfully utilized for dot immunoassays^34^. Lange and coworkers developed a three-dimensional (3D) microarray using wax-patterned nitrocellulose to simultaneously detect multiple proteins^35^. Inspired by this elegant strategy, wax printed paper-based LFA devices have also been developed as an alternative approach for running immunoassays^36^. In this work, wax patterns were printed onto a NC membrane to produce delayed channels that can improve LFA sensitivity by prolonging the incubation time. Generally, the wax printing method requires heating (>120:°C) to melt the wax so that it penetrates into the NC membrane to generate hydrophobic barriers^34–36^. Unfortunately, the pore structure of the NC membrane may be damaged during heating, which leads to poor wettability. More importantly, wax-based barriers can be breached by the surfactant solutions and organic solvents that are generally utilized for bioanalysis^37^. To better meet the requirement of μPADs for immunoassays, it is necessary to develop a reliable approach to fabricate μPADs in NC membranes that are particularly suitable for bioanalysis.

Polyurethane acrylate (PUA), which is a novel photosensitive resin, has been increasingly applied in screen-printing inks, paper glazing coatings and other industrial fields because this material combines the merits of polyurethane and polyacrylate, such as chemical resistance, flexibility and transparency^38,39^. Here, we demonstrate a method to fabricate μPADs by the partial hydrophobization of the NC membrane using PUA. The performance of the proposed μPADs to resist surfactant solutions and organic solvents was evaluated. Furthermore, we designed and assembled a two-layer rotational paper-based analytical device for conveniently implementing multiplexed ELISAs. As a proof of concept, alpha fetoproteins (AFPs) and carcinoembryonic antigens (CEAs) were simultaneously detected on a rotational paper-based analytical device.

## Results and discussion

### Fabrication of the paper-based devices in NC membranes

**Fig. 1:**
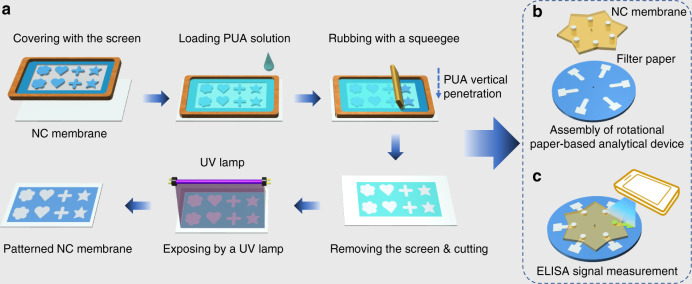
Schematic illustration of the paper-based analysis device. **a** Patterning PUA in the NC membrane. **b** Structure of a rotational paper-based analytical device. **c** The ELISA signal was measured through a portable reader

In this paper, we proposed the use of water-based PUA as a hydrophobic material to fabricate μPADs in NC membranes via screen printing. The whole fabrication process is fast and easy. As shown in Fig. 1a, the PUA solution was first rubbed through the patterned screen and penetrated to the bottom of the NC membrane. After evaporation of the water at ambient temperature, the PUA deposited on the nitrocellulose surface was cured under UV light to form a hydrophobic barrier. Then, a rotational paper-based analytical device for multiplexed ELISAs was prepared by simply assembling the patterned NC membrane and chromatography paper with a hollow rivet (Fig. 1b). The ELISA signals were measured using a portable in-house colorimetric readout device (Fig. 1c). Compared with the previous wax printing method (Table S1), the developed method only needs a low-cost screen stencil and printing table and not an expensive wax printer, and the price of PUA ($8/500 g) is also much lower than that of commercial wax printing ink. In addition, this strategy avoids the problem of the NC membrane sticking in the wax printer due to electrostatic adsorption. Furthermore, the fabrication process does not require heating, avoiding porosity destruction of the NC membrane.

**Fig. 2:**
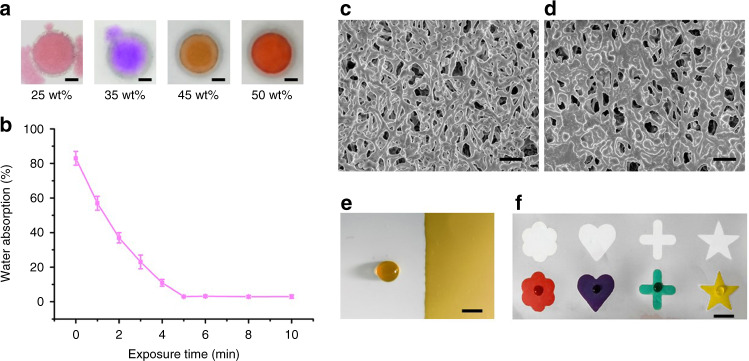
Parameter optimization and characterization of the PUA-patterned NC membranes. **a** Optimization of the concentration of PUA solution. **b** Effect of the exposure time on the water absorption (*n* = 3). **c** SEM image of an untreated NC membrane. **d** SEM image of a PUA-treated NC membrane. **e** Dyed water was dropped into the hydrophobic region (left side) and the hydrophilic region (right side). **f** Dyed water was dropped into the different hydrophilic patterns. **c**, **d** Scale bar = 200 μm. **a**, **e**, **f** Scale bar = 3 mm

The PUA concentration and the exposure time can affect the formation and performance of hydrophobic patterns in the NC membrane. Therefore, we investigated these two control parameters during fabrication. First, hydrophobic circles (6 mm outer diameter, 5 mm inner diameter) were fabricated in the NC membrane using PUA solutions with different concentrations. Figure 2a shows that the dyed water was completely contained by the circles without any leakage when the PUA concentration was increased to 45 wt% but not at lower concentrations. The form of the hydrophobic patterns in the NC membrane was attributed to the crosslinking of PUA. The crosslinking is the result of the radical polymerization of C=C bonds between different PUA molecules under UV light exposure^15^. We further evaluated the effect of exposure time on the crosslinking by testing the water absorption of the PUA-treated NC membrane (water absorption testing was carried out in accordance with a previous method^40^). Figure 2b shows the water absorption of the PUA-treated NC membrane in contact with water after UV exposure times of 0–10 min. It was observed that water absorption decreased gradually with increasing exposure time and then remained constant after 5 min. This result demonstrated that PUA had completely crosslinked and that the NC membrane became hydrophobic. In subsequent work, a PUA concentration of 45 wt% with 5 min UV exposure was chosen to fabricate hydrophobic patterns in the NC membrane.

### Characterization of the paper-based devices in NC membranes

The morphologies of the NC membranes before and after treatment with PUA were illustrated by SEM (Fig. 2c, d). It was found that the porosity of the NC membrane was retained after adsorbing PUA, which indicated that PUA was uniformly deposited on the surface of individual nitrocellulose to form a hydrophobic layer rather than blocking the pores. Microfluidic barriers can function either by transforming cellulose surfaces to be nonwetting or by filling the pores with insoluble materials^41^. From the SEM observations, our strategy adopted the former function. The e containing water solution performance of the PUA-treated NC membrane was evaluated by dropping dyed solution into the hydrophobic and hydrophilic regions of the NC membrane. From Fig. 2e, we can see that the yellow dyed water kept as a drop in the hydrophobic region (i.e., water contact angle of 117°, Fig. S4) but rapidly wetted the whole hydrophilic region. Furthermore, hydrophilic reservoirs with different patterns were fabricated in the NC membrane, and dyed water was pipetted into them. The results showed clear boundaries between the PUA-treated and untreated regions, and the dyed water was completely confined in the reservoirs without leakage (Fig. 2f). Therefore, we concluded that the proposed method could be used to fabricate paper-based devices with well-defined patterns in the NC membrane.

**Fig. 3:**
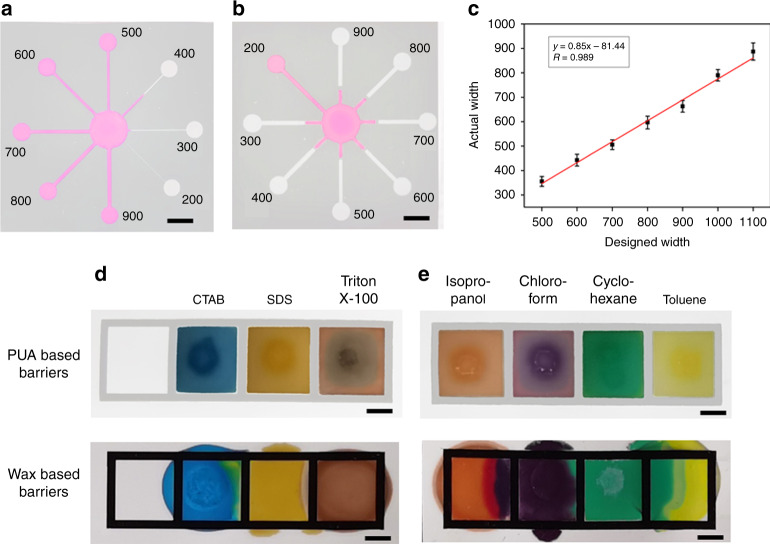
Performance of the paper-based device fabricated in the NC membrane. **a**, **b** The resolution of the PUA-patterned hydrophilic microchannel channels and hydrophobic barriers, respectively. **c** The relationship between the designed width and actual width of the microchannels. Comparison of the PUA-based barriers and the wax-based barriers for resisting the surfactant solutions **d** and the organic solvents **e**. Scale bar = 4 mm

The hydrophilic-hydrophobic pattern resolution in the NC membrane was evaluated in accordance with previously described methods^34^. Briefly, eight microfluidic microchannels and hydrophobic barriers with increasing widths (from 200 to 900 μm, in increments of 100 μm) were fabricated in the NC membrane. The dyed water was loaded in the middle circle reservoirs and flowed outward under capillary action. The actual widths of the microchannels and barriers were measured using a digital microscope (Olympus DSX510). It can be seen from Fig. 3a that the thinnest microchannel that enabled the aqueous solution to flow into the terminal reservoir was 500 μm in designed width (measured width was 356 ± 25 μm; *n* = 3). The reason for the reduced width of the measured microchannel is that the PUA solution not only penetrates through the NC membrane vertically but also spreads laterally during screen printing. As shown in Fig. 3b, the thinnest hydrophobic barrier that could prevent aqueous solution flow was 300 μm in designed width (measured width was 321 ± 20 μm). *n* = 3). Thinner barriers were unable to resist the penetration of the aqueous solution. The relationship between the designed width and measured width of the microchannels was also investigated. As illustrated in Fig. 3c, the measured width was linearly dependent upon the designed width with a good correlation coefficient of 0.989 (*n* = 3). It is anticipated that the microchannels in the NC membrane can be precisely controlled and reproducibly constructed using the developed method.

Previous reports have demonstrated that wax-based barriers can be destroyed by surfactant solutions and organic solvents, which restricts their applications in bioassays^15,40^. To evaluate the ability of PUA-based barriers to confine aggressive liquids, different surfactant solutions (1 wt% CTAB, SDS and Triton X-100) and organic solvents (isopropanol, chloroform, cyclohexane and toluene) were pipetted into square patterns (size: 6 × 6 mm, PUA barrier width: 1 mm) in the NC membrane. For comparative research, wax-based square patterns with the same sizes were also fabricated in the NC membrane using the wax printing method^42^. Figure 3d shows that the PUA-based barriers were capable of containing all surfactant solutions, while the wax-based barriers failed to support these surfactant solutions with serious leakage through the hydrophobic barrier. Figure 3e shows the robustness of the PUA-based barriers when they contained various organic solvents. It was found that none of the organic solvents could breach the PUA-based barriers. On the other hand, the wax-based barriers could not resist any of the organic solvents. The excellent ability of the PUA-based barriers to resist surfactant solutions and organic solvents is because the formed crosslinked polymer on the nitrocellulose surface was hardly dissolved by the solutions and solvents. Hence, the paper-based device fabricated in the NC membrane using the proposed method possesses more versatile applications than those using the wax printing method and is especially suitable for complex biochemical reactions and bioanalyses that require surfactant solutions or organic solvents.

### Rotational paper-based analytical device assay procedure

**Fig. 4:**
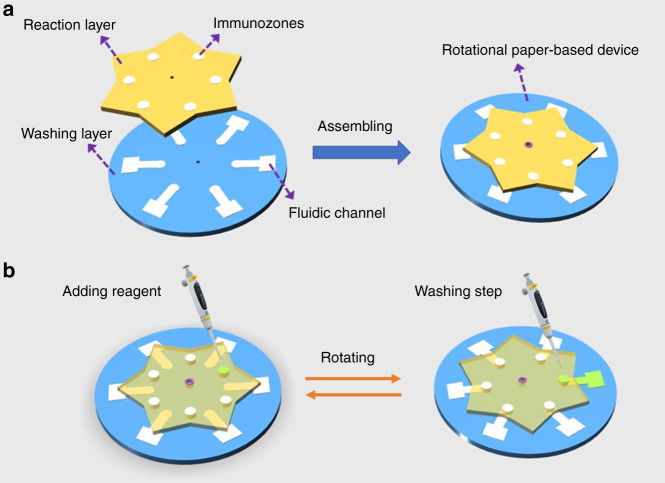
Rotational paper-based analytical devices. **a** Design and assembly of the rotational paper-based analytical devices. **b** Operation of the adding and washing steps during an ELISA reaction. First, the sample or reagent was added to the immunozone for incubation (valve OFF). Then, the top piece was rotated by 30° to connect the immunozone and the washing channel of the bottom piece (valve ON), and unbound reagent was rinsed away along the washing channel

A typical ELISA experiment requires multiple steps of incubation and washing in a specific order before detection. In this work, we integrated a complex immunoassay operation on a portable and user-friendly rotating paper-based device. As a proof of concept, two cancer biomarkers, AFP and CEA, were detected on this assay platform. As shown in Fig. 4b, the immunoassay process could be implemented by manually controlling the “rotary valve”. For the incubation step, the top piece was rotated counterclockwise by 30° to disconnect the immunozones on the reaction layer and the flow channels on the washing layer (valve OFF), and the subsequent reagents were added to the immunozones for incubation. While the washing step was performed, the top piece was rotated clockwise by 30° to connect the immunozones and the washing channels (valve ON), and unbound reagents were rinsed away along the washing channels. The entire ELISA procedure for AFP and CEA was established on the rotational paper-based analytical device via orderly incubation of the capture antibody, antigen, signal antibody, and several washes. Based on the discussion above, it is clear that this rotating paper-based device can carry out multistep immunoassays in a facile and fast manner.

### Optimization of ELISA conditions

**Fig. 5:**
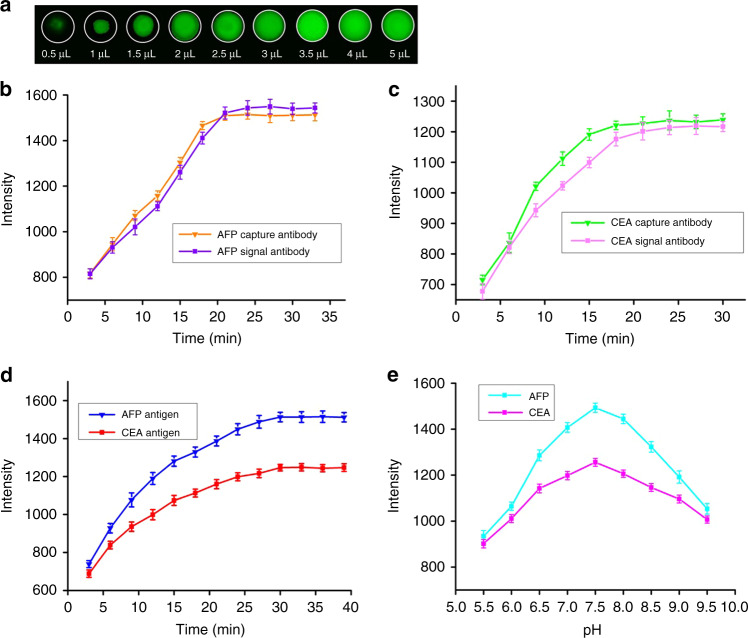
Effect of the experimental parameters for ELISA signals on the rotational paper-based analytical device. **a** Optimization of pipetting volume loaded into the reaction zone. **b**–**d** The effect of the incubation times (capture antibody, signal antibody and antigen) on the signal intensity. **e** The effect of pH on the signal intensity (*n* = 3)

Sample consumption plays a dominant role in the reduction of assay cost. First, we optimized the volume of solution loaded into the immunozones of the rotational paper-based analytical device. 40 ng/mL FITC-labeled CEA antibody solutions with different volumes were added to the immunozones and observed with an inverted fluorescence microscope (Olympus IX51). As illustrated in Fig. 5a, the antibody solution evenly filled the entire immunozone after the pipetting volume increased to 4 μL. Consequently, we selected the optimal pipetting volume as 4 μL. BSA was used to block the residual protein-binding site of immunozones, which is important to eliminate nonspecific adsorption. We investigated a series of concentrations of BSA solution that were dropped into the immunozones (detailed experimental steps are provided in the Supporting Information). As shown in Fig. S5, when the concentration of BSA was 0.5%, the protein-binding sites in the immunozones were completely blocked. To ensure complete capture of the antigens, we optimized the concentration of the captured antibody (Fig. S6). Based on the test results, 10 μg/mL was chosen as the concentration for the capture antibodies. Analysis time is critical for POCT, and we further investigated the influence of the incubation times of the capture antibody and HRP-labeled antibody on the detection signal intensity. Figure 5b, c shows that the signal intensity for the captured antibody of AFP and CEA reached their plateau at 21 min, and the signal intensity for the HRP-labeled antibody of AFP and CEA reached their plateau at 24 min. The incubation time of the AFP and CEA antigen was also evaluated, and the data indicated that the optimum incubation time was 30 min (Fig. 5d). The pH of the solution significantly affects the activity of the immobilized proteins. As presented in Fig. 5e, the signal intensity increased when the pH increased from pH 5.0 to 7.5, followed by a decrease until pH 9.5, which meant that the maximum signal intensity was achieved at approximately pH 7.5. Accordingly, we chose PBS with a pH of 7.5 for the experiments discussed below.

### Analytical performance of the rotational paper-based analytical device

**Fig. 6:**
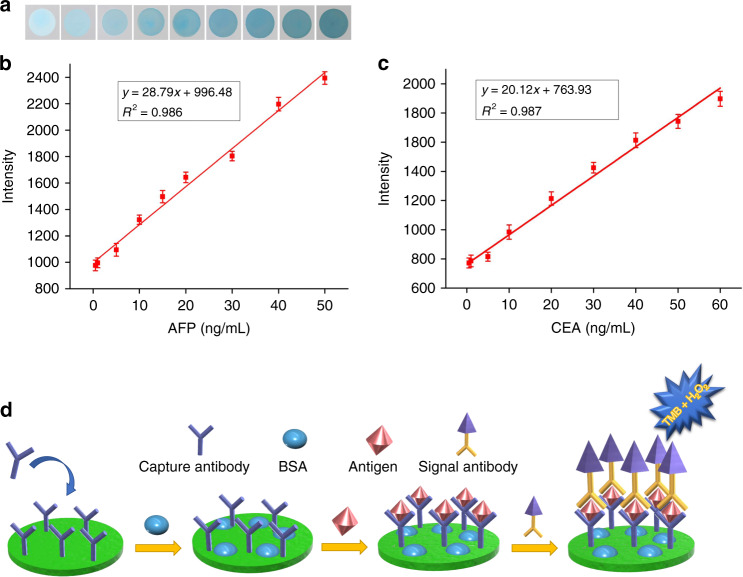
Evaluation of performance. **a** Photographic images of the immunozones after loading the (concentrations between 0 and 50 ng/mL; 0.5, 1, 5, 10, 15, 20, 30, 40 and 50 ng/mL). **b** Calibration curves for the determination of AFP and (**c**) CEA (*n* = 3). **d** Schematic diagram of the ELISA procedure on the rotational paper-based analytical device

To further validate the multiplexed ELISA performance of the rotational paper-based analytical device, six sandwich HRP-labeled immunoassays were simultaneously performed to detect two tumor markers (AFP and CEA) (Fig. S7). The ELISA step on this device is shown in Fig. 6d. Using TMB-H_2_O_2_ solution to trigger a chromogenic reaction, a blue color appeared on the reaction layer.

Under the optimal conditions, AFP and CEA standard solutions with different concentrations were detected. As shown in Fig. 6a, with increasing concentrations of AFP, the colorimetric intensity gradually increased. A good linear correlation was obtained over the concentration ranges of 0.5–50.0 ng/mL for AFP (Fig. 6b) and 0.5–60.0 ng/mL for CEA (Fig. 6c). The linear regression equations were *y* = 28.79*x* + 996.48 (*R*^2^ = 0.986) and *y* = 20.12*x* + 763.93 (*R*^2^ = 0.987), respectively, where *y* is the relative intensity and *x* is the concentration of the corresponding biomarker. According to LOD = 3σ/S, where σ is the standard deviation of the intensity from the blank samples and S is the slope of the linear fitting curve, the LOD was calculated to be 136 pg/mL for AFP and 174 pg/mL for CEA. The threshold limits of these two cancer biomarkers in clinical diagnosis are 25 ng/mL and 5 ng/mL, respectively^25^. Hence, the proposed device presents a potential POCT platform to sensitively detect two cancer biomarkers.

For a new ELISA strategy, selectivity must be considered. To evaluate the selectivity of the proposed device for AFP and CEA analysis, some possibly interfering biomarkers in human serum were investigated, such as PSA, CRP and PCT. As shown in Fig. S8A, the colorimetric intensity of the AFP solutions mixed with other biomarkers was almost the same as that of the pure AFP solution. In contrast, the signal responses of the separated interfering biomarkers were negligible for AFP. Similar results were also observed for CEA analysis (Fig. S8B). Thus, such good selectivity indicated that the proposed device was feasible for practical applications.

### Clinical diagnostic application

The clinical applicability of the rotational paper-based analytical device was demonstrated via assays of AFP and CEA in clinical serum samples, and the results were compared to those provided by Yantai Affiliated Hospital of Binzhou Medical University. As listed in Table S2, both positive and negative samples were detected using the proposed device, and the results were almost consistent with the standard hospital detection methods. It was also observed that the relative standard deviation (RSD) of the data from this device ranged from 1.2% to 4.7%, demonstrating acceptable reliability. Therefore, the rotational paper-based analytical device was capable of monitoring the levels of cancer biomarkers in clinical samples.

## Conclusion

We proposed a simple and inexpensive method to fabricate μPADs in a NC membrane. To the best of our knowledge, this is the first demonstration of μPADs using a screen-printing technique combining UV-curing PUA to pattern hydrophobic barriers. More importantly, the resulting PUA barriers presented excellent resistance to the surfactant solutions and organic solvents that are critical for complex biochemical reactions and bioanalysis on paper-based platforms. Based on the developed fabrication method, we further constructed a rotational paper-based analytical device for implementing multiplexed ELISA experiments in a simple manner. As a proof-of-concept application, quantitative analysis of AFP and CEA was successfully performed on this device with line ranges of 0.5–50.0 ng/mL and 0.5–60.0 ng/mL, respectively. Consequently, the proposed rotational paper-based analytical device is a highly promising tool for biomarker analysis and can be readily adapted for POCT or in resource-poor settings. We also expect that this device can be extended to more application fields, including protein-related diagnosis, environmental monitoring and food safety.

## Materials and methods

### Materials

NC membranes (CN-SH34, 0.45 μm pore size) were obtained from MDI (Ambala Cantt, India), and Whatman No. 1 chromatography paper was purchased from GE Healthcare Worldwide (Pudong Shanghai, China). Water-based PUA containing 45 wt% prepolymer was obtained from Nanjing Jiazhong Chemical Technology Co., Ltd. (Nanjing, China). Isopropanol, chloroform, cyclohexane, toluene, sodium dodecyl sulfate (SDS) and 2-hydroxy-2-methylpropiophenone (HMPP) were obtained from Sinopharm Chemical Reagent (Shanghai, China). Cetyltrimethylammonium bromide (CTAB) and Triton X-100 were obtained from Sigma-Aldrich. AFP, CEA, AFP and CEA capture antibody, HRP-labeled AFP and CEA signal antibody, C-reactive protein (CRP), prostate specific antigen (PSA) and procaicitonin (PCT) were purchased from Shanghai Linc-Bio Science Co. Ltd. Phosphate buffered-saline (PBS; 10 mM, containing 0.05% Tween-20, pH = 7.5) was used as the washing buffer. Ultrapure water was produced with an Aquaplore 2C intelligent laboratory water purification system and used throughout the whole experiment. Human serum samples were obtained from Yantai Affiliated Hospital of Binzhou Medical University with ethical review and approval (project identification code: IRB2020-106).

### Fabrication of the paper-based devices in NC membranes

A representation of the fabrication process for the paper-based devices in the NC membrane is shown in Fig. 1a and Fig. S1. The hydrophilic pattern was designed with Adobe Illustrator software and transferred to a screen stencil (400 meshes of polyester) with a wooden frame. First, the NC membrane was laid on the printing table and covered with the patterned screen stencil. Next, the mixed solution of water-based PUA and photoinitiator HMPP (3% of the total mass of PUA formulation) was loaded on the screen stencil and then evenly rubbed through the screen surface using a squeegee. The mixed solution could penetrate into the interior of the NC membrane through the pore. After drying at ambient temperature for 30 min, the NC membrane was exposed to a UV lamp (LED, 365 nm, 15 mW/cm^2^) for 5 min to cure the PUA; after this, it was ready for use. The screen stencil was washed with water to remove the residual PUA for the next printing.

### Design and assembly of the rotational paper-based analytical device

As shown in Fig. 4a and Fig. S2, the rotational paper-based analytical device was designed to consist of two layers, a reaction layer (top piece) and a washing layer (bottom piece). The reaction layer contained six immunozones (6 mm diameter) for loading samples and reagents, which were fabricated in the NC membrane using the proposed method. The washing layer contained six fluidic channels (40 mm long) that connect to the immunozones on the reaction layer to implement the washing process. This layer was fabricated using Whatman No. 1 chromatography paper (which can contain larger volumes of washing buffer and is much less expensive than NC membranes) via the wax printing method as described in a previous report^42^. The assembly process was as follows. Briefly, the reaction layer and the washing layer were stacked in order, and then a hollow rivet was punched through the center hole of each layer. Finally, the end of the hollow rivet was hit and expanded with a hammer to assemble these two layers together. The developed hybrid paper-based device can be freely rotated to achieve the switch between washing and incubation processes during ELISA. Furthermore, it should be noted that six sets of ELISA experiments can be simultaneously performed on this device, which presents a potential application in multiplexed analysis. The diameter of this portable device was 100 mm, and the reaction layer was designed as a hexagram to facilitate the location of the immunozones and washing channels.

### ELISA procedure on the rotational paper-based analytical device

Using the cancer markers AFP and CEA as target analytes, multiplexed ELISAs were performed on the proposed rotational paper-based analytical device. First, 4 μL of 10 μg/mL AFP and CEA capture antibodies were dropped into the three groups of immunozones at intervals. After incubation at ambient temperature, the unbound capture antibodies were removed by six washes. Subsequently, 4 μL of 0.5% BSA solution was dropped into each immunozone to block the remaining protein-binding site. After that, 4 μL of AFP and CEA standard solutions were dropped into the immunozones and allowed to be incubated at ambient temperature with the same washing step. Then, 4 μL of 10 μg/mL HRP-labeled signal antibodies was added dropwise, incubated and washed sequentially. Finally, 3 μL of 3,3′,5,5′-tetramethylbenzidine (TMB)-H_2_O_2_ solution was spotted into the immunozones to trigger the chromogenic reaction. After 3 min, the colorimetric intensities were measured using our in-house-made colorimetric readout device (Fig. S3)^43^.

## Supplementary information

Supporting information
