# Supplementary material for: A novel polymer-based nitrocellulose platform for implementing a multiplexed microfluidic paper-based enzyme-linked immunosorbent assay
Source: Microsyst Nanoeng. 2022 May 19;8:53. doi: 10.1038/s41378-022-00385-z (PMC9120459; doi:10.1038/s41378-022-00385-z)
Supplement: Supplementary file 1 — Supporting information [file 41378_2022_385_MOESM1_ESM.docx]

Supporting Information

**A Novel Polymer-based Nitrocellulose Platform for Implementing Multiplexed Microfluidic Paper-based** **Enzyme-Linked Immunosorbent Assay**

Dong Lin ^1,2,6,#^, Bowei Li ^1,#,^*, Longwen Fu ^1^, Ji Qi ^1,5^, Chunlei Xia ^1^, Yi Zhang ^3^, Jiadong Chen ^4^, Jaebum Choo ^4^*, and Lingxin Chen ^1,2,5^*

*1 CAS Key Laboratory of Coastal Environmental Processes and Ecological Remediation; Shandong Key Laboratory of Coastal Environmental Processes, Yantai Institute of Coastal Zone Research, Chinese Academy of Sciences, Yantai 264003, China*

*2 School of Pharmacy, Binzhou Medical University, Yantai 264003, China*

*3 Institute of Analytical Food Safety, School of Food Science and Technology, Jiangnan University, Wuxi 214122, China*

*4 Department of Chemistry, Chung-Ang University, Seoul 06974, South Korea*

*5 Center for Ocean Mega-Science, Chinese Academy of Sciences, Qingdao 266071, China*

*6 University of Chinese Academy of Sciences, Beijing 100049, China*

*Corresponding Authors.

Phone: +86-535-2109130. Fax: +86-535-2109130.

E-mail addresses: bwli@yic.ac.cn (B. Li), jbchoo@cau.ac.kr (J. Choo), lxchen@yic.ac.cn (L. Chen)

^#^ Dong Lin and Bowei Li contributed equally to this work.

**List of Contents:**

1. Figure S1. The actual fabrication process for the hydrophobic barriers in the NC membrane
2. Figure S2. Photograph of the rotational paper-based device
3. Figure S3. Photograph of our in-house made colorimetric readout device
4. Figure S4 Water contact angle of the PUA treated NC membrane.
5. Figure S5 Fluorescence images of the immunezones after applying different concentrations of BSA solution.
6. Figure S6 The effecting of the capture antibody concentration on the signal intensity.
7. Figure S7. Chromogenic response in immunozones after loading TMB-H_2_O_2_ solution
8. Figure S8 Selectivity of the proposed paper-based device for detection of AFP and CEA
9. Table S1 Comparison of fabrication processes and features between the proposed method and wax printing method
10. Table S2. Assay results of twelve human serum samples from both the rotational paper-based analytical device and the standard hospital method.


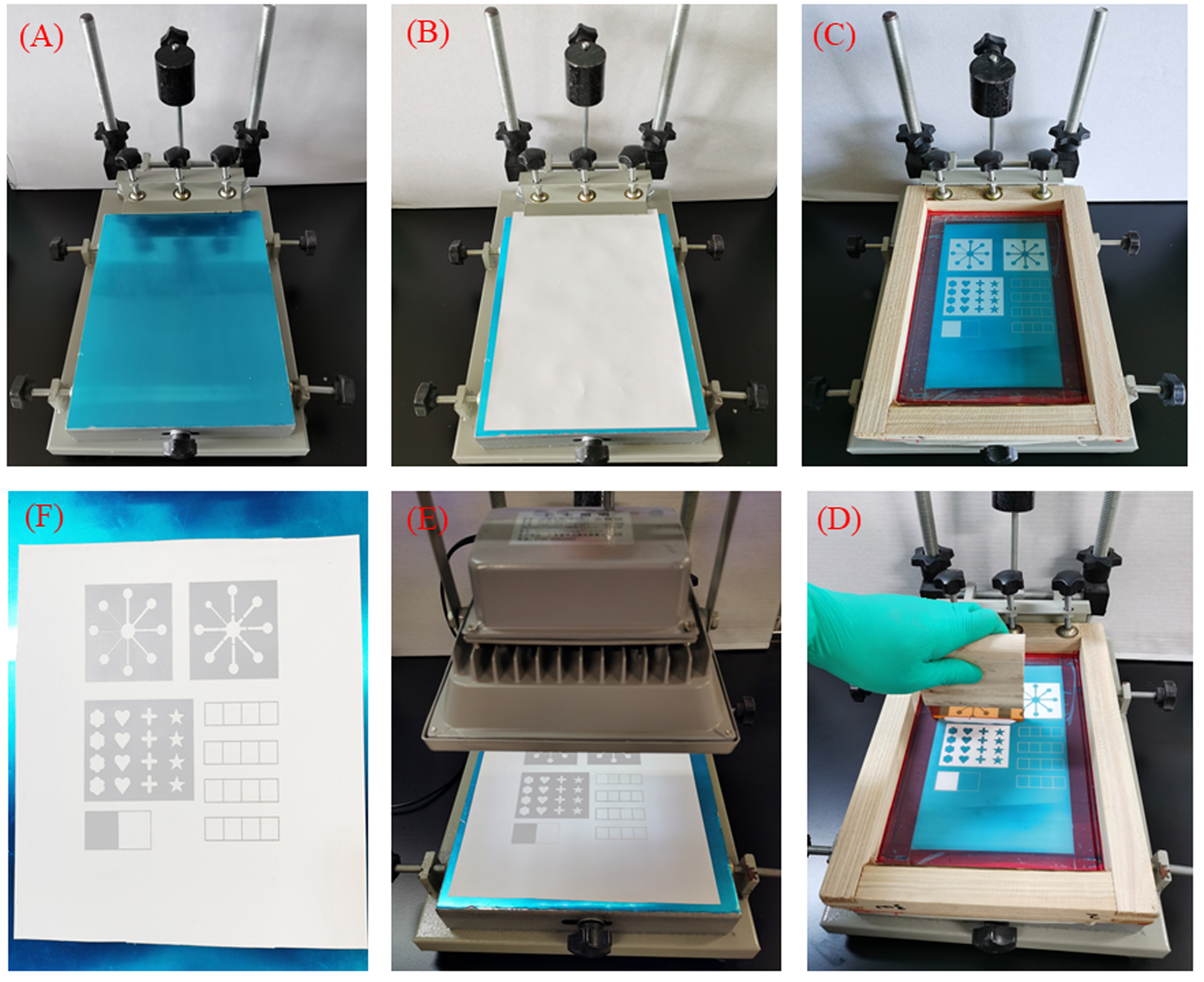


**Fig. S1.** The actual fabrication process for the hydrophobic barriers in the NC membrane.

(A) Printing table; (B) NC membrane was laid on the printing table; (C) The NC membrane was covered with a patterned screen stencil; (D) PUA solution was evenly rubbed through the surface of the screen stencil; (E) the PUA treated NC membrane was exposed by a UV lamp; (F) The patterned NC membrane.


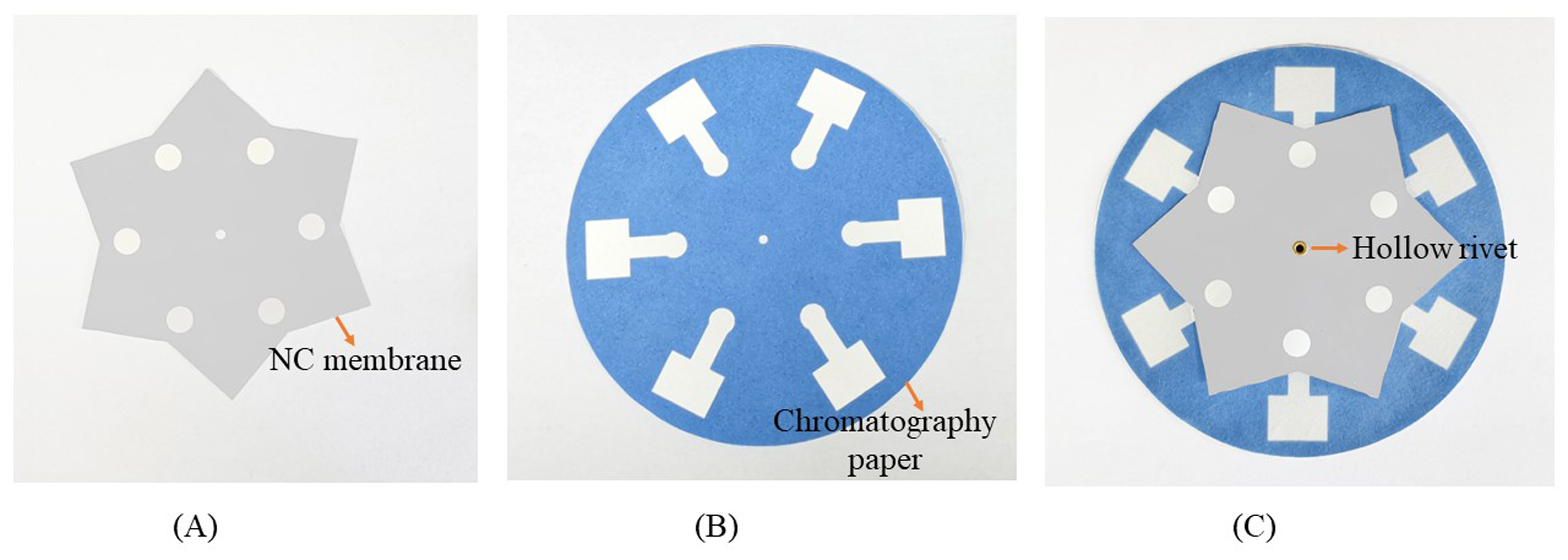


**Fig. S2.** Photograph of the rotational paper-based device. (A) Reaction layer prepared in the NC membrane though screen printing PUA. (B) Washing layer prepared in the Whatman No.1 chromatography paper through wax printing method. (C) A hybrid paper-based device was constructed via using a hollow rivet to assemble reaction layer and washing layer together.


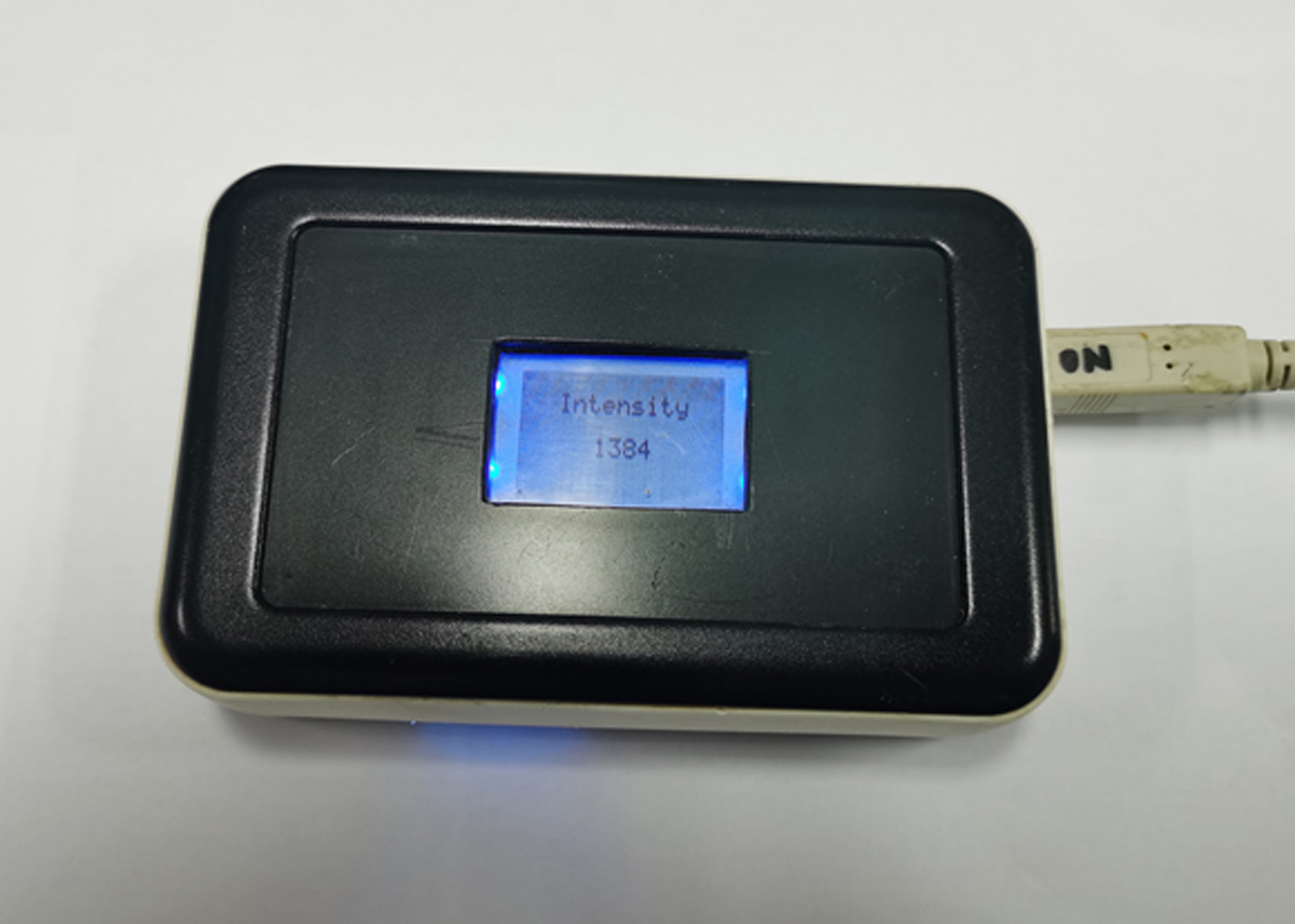


**Fig. S3** Photograph of our in-house made colorimetric readout device


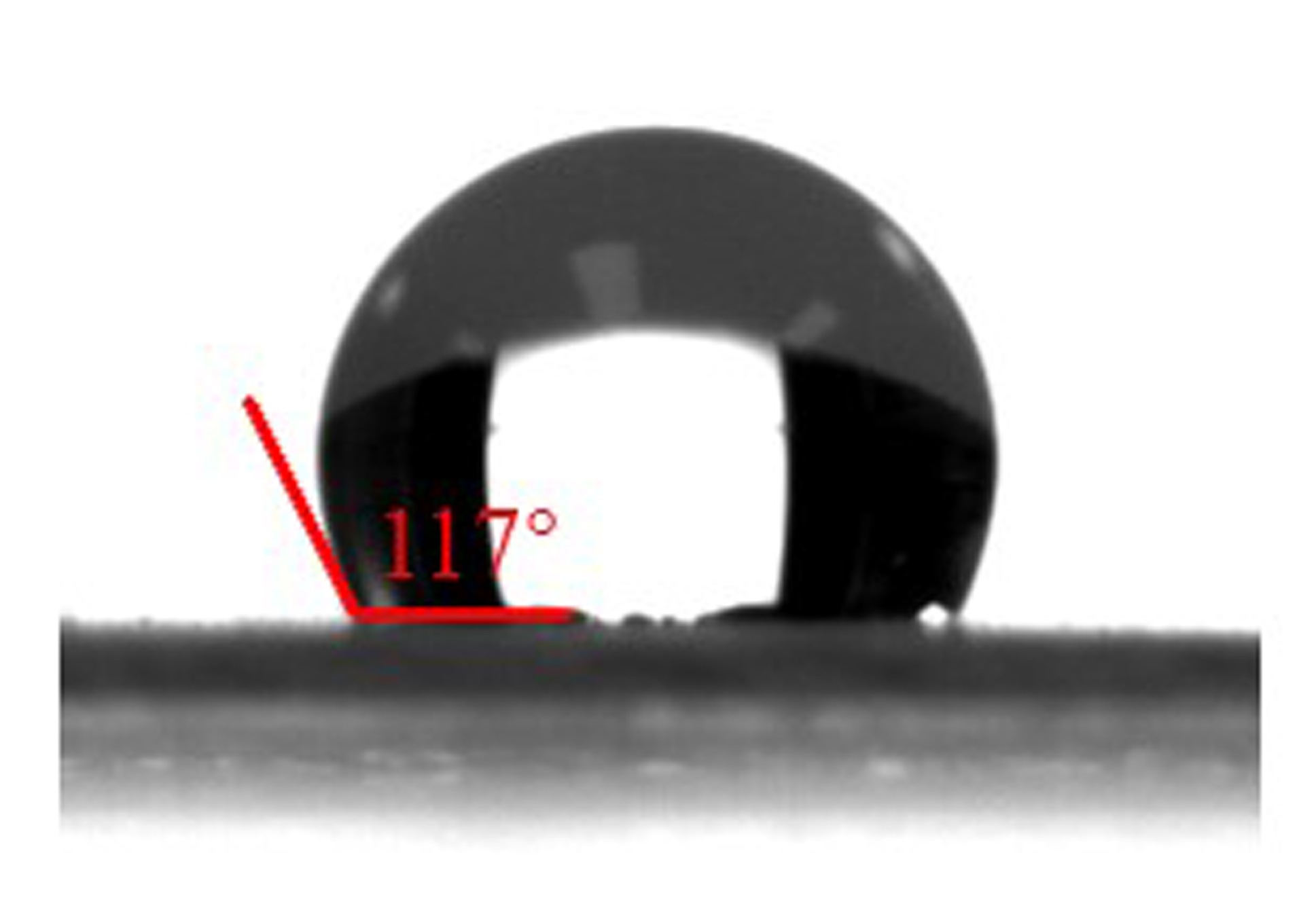


**Fig. S4** Water contact angle of the PUA treated NC membrane.


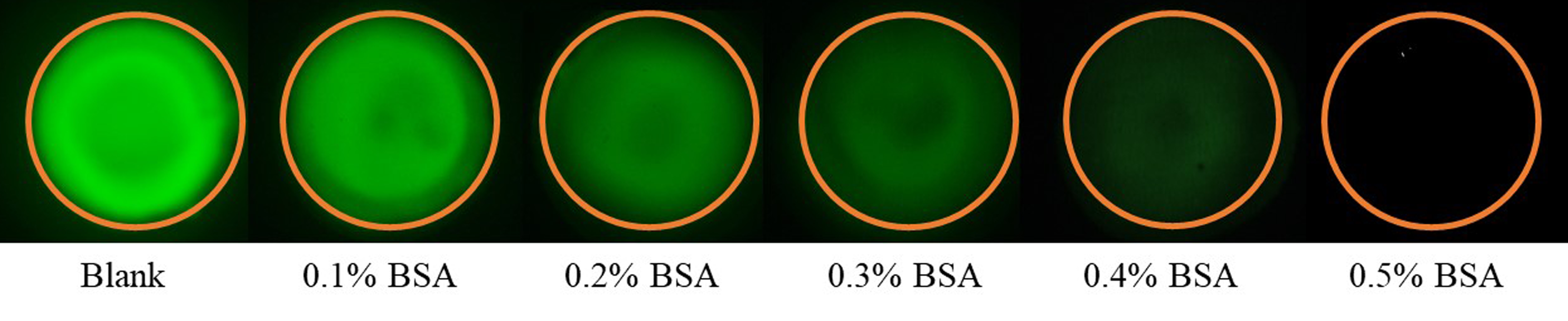


**Fig. S5** Fluorescence images of the immunezones after applying different concentrations of BSA solution. Firstly, 4 μL of different concentrations (from 0.1% to 0.5%) of BSA solution were loaded in immunezones and incubation for 10 min at ambient temperature. Then the immunezones was washed six times. Next, 40 ng/mL FITC-labeled CEA antibody solution was added into the immunezones and incubation for 30 min, following by the same washing step. Finally, the immunezones were observed with an inverse fluorescence microscope (Olympus IX51).


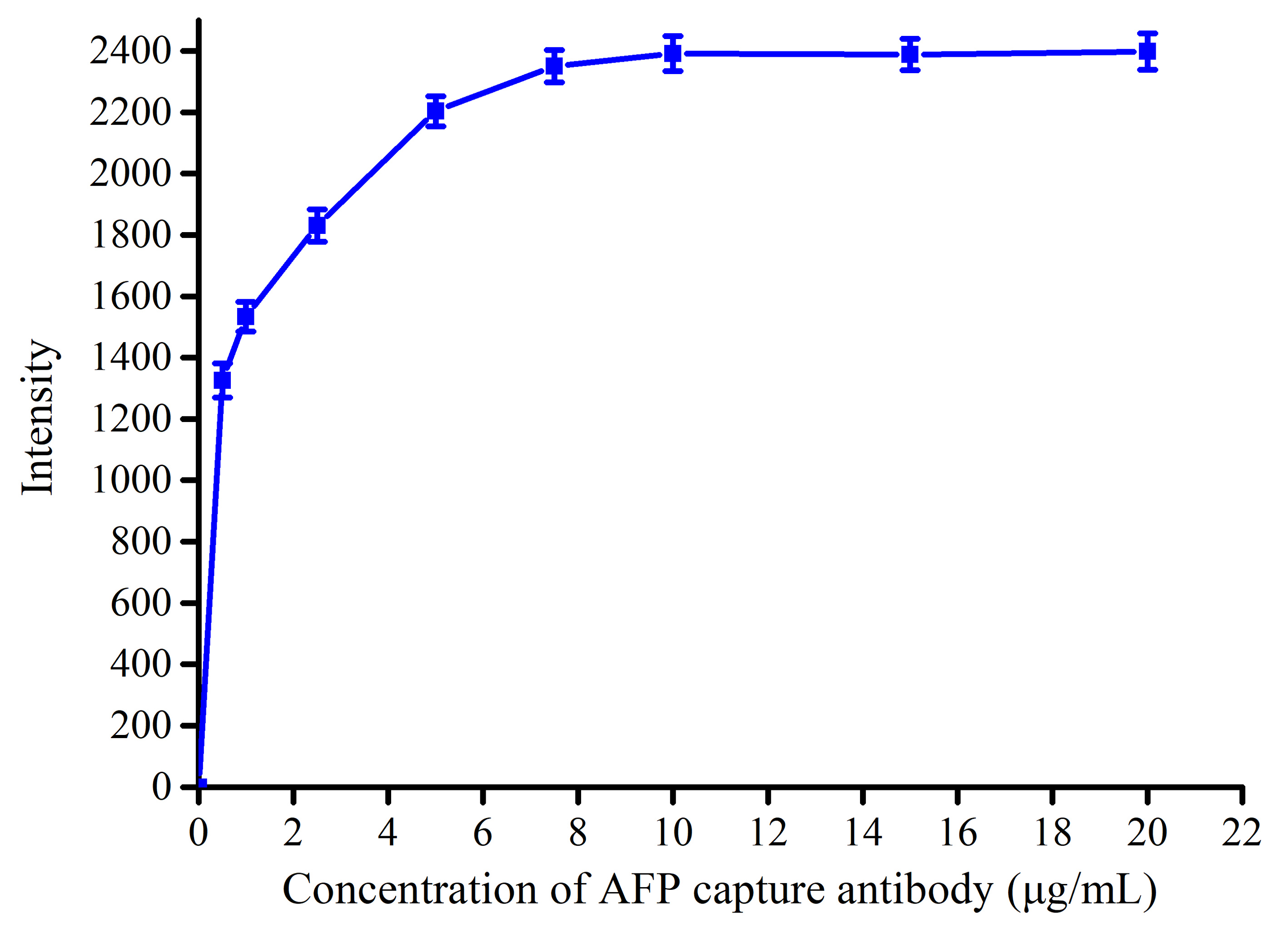


**Fig. S6** The effecting of the capture antibody concentration on the signal intensity (n = 3). Using 50 ng/mL of AFP and 10.0 μg/mL of HRP-labeled AFP signal antibody, the concentrations of the capture antibody were tested from 0.5 to 20 μg/mL.


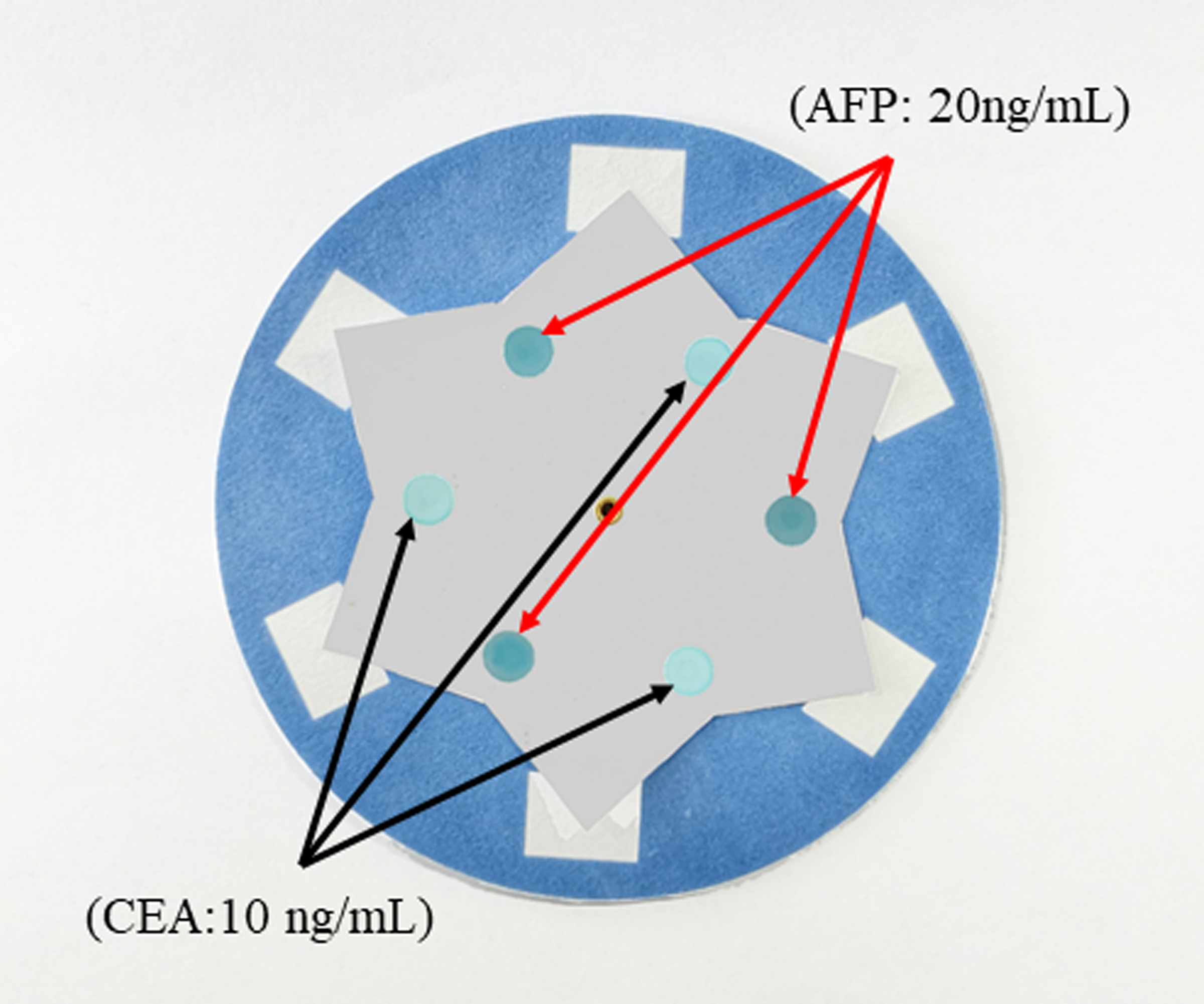


**Fig. S7** Chromogenic response in immunozones after loading TMB-H_2_O_2_ solution


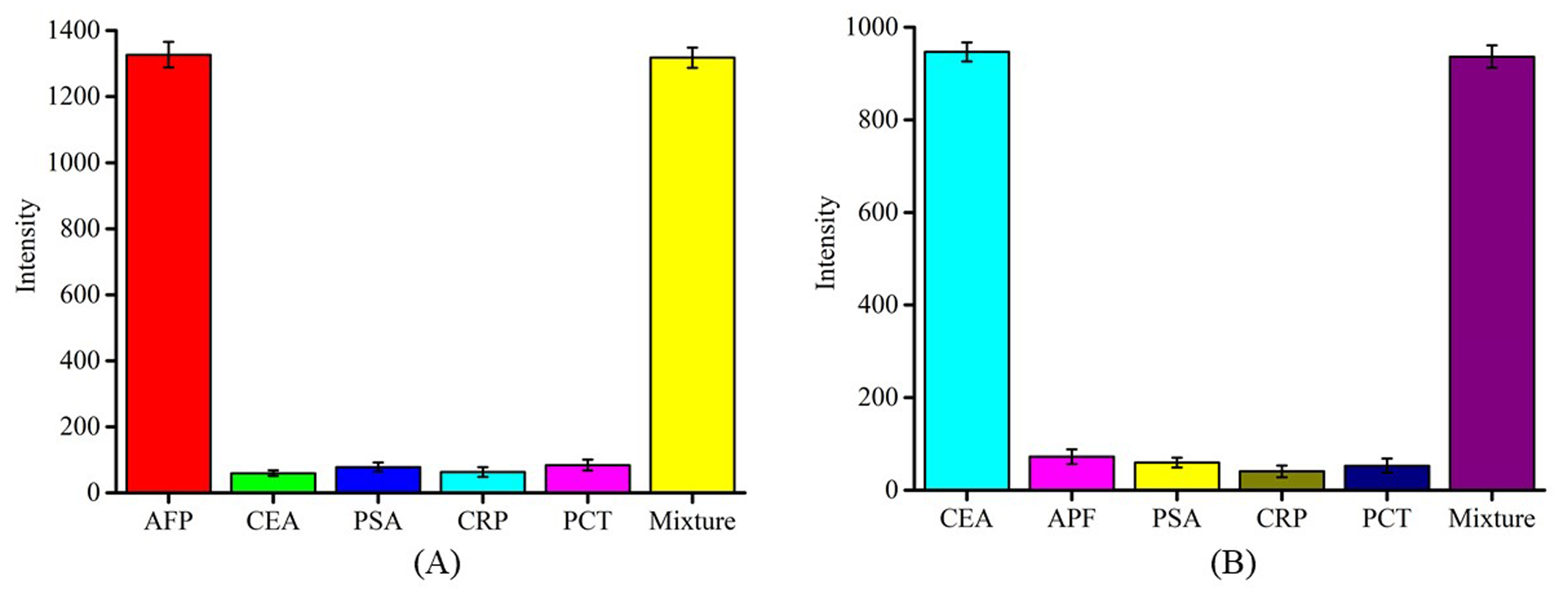


**Fig. S8** Selectivity of the proposed paper-based device for detection of AFP (A) and CEA (B) (n = 3).

**Table S1** Comparison of fabrication processes and features between the proposed method and wax printing method.

| Method | Equipment prices ($) | Material price (500g, $) | Rapid prototyping | Chemical resistance |
| --- | --- | --- | --- | --- |
| Proposed method | ~20 | ~8 | Yes | Supporting surfactants and organic solvents |
| Wax printing method | 800-1000 | 260~300 | Yes | No surfactants and organic solvents |

1. **Table S2** Assay results of twelve human serum samples from both the rotational paper-based analytical device and the standard hospital method (n = 3).

(n = 3)

**^#^**The data of the hospital standard detection method were provided by Yantai Affiliated Hospital of Binzhou Medical University.

| Patient 12 | 31.35±1.03 | 31.43 | 2.0 | 53.71±1.48 | 53.87 | 1.6 |
| --- | --- | --- | --- | --- | --- | --- |
| Patient 11 | 39.88±1.20 | 39.65 | 1.8 | 10.09±0.34 | 10.16 | 1.7 |
| Patient 10 | 29.37±0.86 | 29.46 | 1.7 | 35.29±0.88 | 35.03 | 1.5 |
| Patient 9 | 47.61±1.21 | 47.83 | 1.5 | 21.53±0.84 | 21.70 | 2.3 |
| Patient 8 | 42.15±0.86 | 42.38 | 1.2 | 4.91±0.17 | 4.77 | 2.0 |
| Patient 7 | 36.86±1.30 | 36.51 | 2.1 | 2.43±0.12 | 2.56 | 2.9 |
| Patient 6 | 2.27±0.1 | 2.36 | 2.2 | 4.30±0.18 | 4.46 | 2.1 |
| Patient 5 | 14.55±0.48 | 14.20 | 1.3 | 13.63±0.38 | 13.71 | 1.3 |
| Patient 4 | 3.87±0.13 | 4.06 | 1.6 | 9.63±0.45 | 9.87 | 2.3 |
| Patient 3 | 0.53±0.05 | 0.61 | 4.7 | 1.47±0.11 | 1.23 | 3.7 |
| Patient2 | 3.71±0.15 | 3.62 | 2.0 | 2.07±0.12 | 2.15 | 2.9 |
| Patient 1 | 3.01±0.11 | 2.93 | 1.7 | 0.84± 0.06 | 0.75 | 3.0 |
| Method | Proposed method | Hospital methods**^#^** | RSD (%) | Proposed method | Hospital methods | RSD (%) |
| Tumor marker | AFP (ng/mL) | | | CEA (ng/mL) | | |
